# A Strategy Based on Loop Analysis to Develop Peptide Epitopes: Application to SARS-CoV-2 Spike Protein

**DOI:** 10.3389/fmolb.2021.658687

**Published:** 2021-05-05

**Authors:** Maria Luisa Di Vona, Gian Maria Rossolini, Marco Sette

**Affiliations:** ^1^Department of Industrial Engineering and International Associated Laboratory: Ionomer Materials for Energy, University of Rome Tor Vergata, Rome, Italy; ^2^Department of Experimental and Clinical Medicine, University of Florence, Florence, Italy; ^3^Microbiology and Virology Unit, Careggi University Hospital, Florence, Italy; ^4^Department of Chemical Sciences and Technology, University of Rome Tor Vergata, Rome, Italy; ^5^Sorbonne Paris Cité, CSPBAT Laboratory, University of Paris 13, UMR 7244, CNRS, Bobigny, France

**Keywords:** SARS-CoV-2, COVID-19, loop regions, bioconjugate polymers, hybrid protein-polymer

## Abstract

Many current strategies for inducing an immune response rely on the production of an antigenic protein. Such methods can be problematic if the folding of the antigenic protein is incorrect. To avoid this problem, we propose a method based on grafting specific regions of the chosen antigenic protein onto biocompatible polymeric matrices, so that they can mimic portions of the antigenic protein. These regions are selected following the criterion according to which they are not folded, are exposed to the solvent and are not already present in the human body, so that they are not recognized by the immune system as self. Regions are selected using the primary sequence of the protein and, where possible, its tertiary structure. The application of this strategy to the Spike protein of SARS-CoV-2 is presented.

## Introduction

The recent and ongoing COVID-19 pandemic has stressed the importance of adopting strategies for a rapid response against infections.

The search for vaccines uses different methodologies which are well-exemplified by those pursued in the recent pandemic, but all are based on the rationale of preventing viral infection of the human host cells.

**FIGURE 1:**
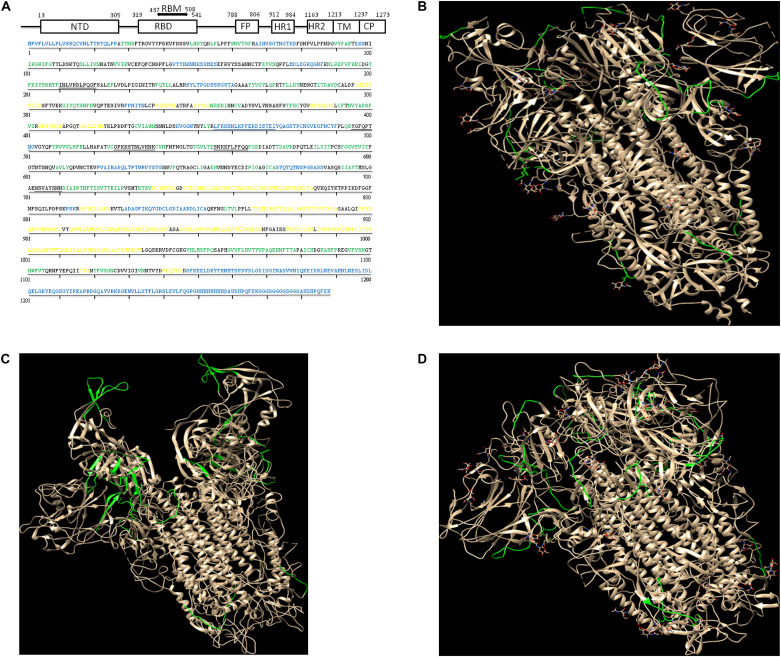
Location of the proposed regions in the Spike protein and comparison with Spike proteins from other coronavirus. **(A)** Top: schematic view of the Spike protein with the specifications of the different domains: NTD, N-Terminal Domain; RBD, Receptor Binding Domain; RBM, Receptor Binding Motif; FP, Fusion Peptide; HR1, Heptad Repeat 1; HR2, Heptad Repeat 2; TM, TransMembrane region; CP, Cytoplasmic Domain. The S1 domain consists of the NTD and RBD regions. Bottom: primary sequence of Spike protein in SARS-CoV-2 in the prefusion state (Wrapp et al., 2020; PDB code 6vsb, chain A). Helices are colored in yellow, sheets are colored in green and the missing regions in the crystal structure are colored in blue. The regions reported in the text are underlined. **(B)** 3D structure of Spike protein in SARS-CoV-2 in the prefusion state (Wrapp et al., 2020; PDB code 6vsb) where the regions reported in the text are colored in green for each monomer. **(C)** 3D structure of Spike protein in MERS-CoV in the prefusion state (Yuan et al., 2017; PDB code 5 × 5c). The regions corresponding to the ones reported in text are colored in green for each monomer. **(D)** 3D structure of Spike protein in SARS-CoV in the prefusion state (Yuan et al., 2017; PDB code 5 × 58). The regions corresponding to the ones reported in text are colored in green for each monomer. The equivalent residues in MERS-CoV and SARS-CoV were obtained by multiple alignment using the ClustalW software (Larkin et al., 2007). The figures were prepared with Chimera software (Pettersen et al., 2004).

In the case of the SARS-CoV-2 and closely related SARS-CoV viruses, responsible of COVID-19 and SARS respectively, the first host cell interaction occurs via bonding between the viral envelope Spike protein and the human ACE2 cell receptor. Spike is a homotrimeric membrane protein consisting of three identical subunits, each one composed of an inner viral region, a transmembrane region, and an extracellular region composed of an S2 domain proximal to the viral envelope and an S1 domain distal to the viral envelope, Figure 1A. The latter domain interacts with the cell receptor through the Receptor Binding Domain (RBD) region (Walls et al., 2020).

Several three-dimensional structures of entire Spike proteins from SARS-CoV and SARS-CoV-2 are known, both in the prefusion state (Yuan et al., 2017; Melero et al., 2020; Walls et al., 2020; Wrapp et al., 2020) and in the bound form with the receptor, as entire protein or RBD domain (Li et al., 2005; Lan et al., 2020). Analysis of the entire structures shows that the Spike proteins are structurally very similar for both viruses, and have a large surface exposed to the solvent, part of which is responsible for the interaction with the receptor. Despite the strong structural similarity (the RBD domains of both proteins can be superimposed with an r.m.s.d. of 1.2 Ǻ, the viral protein sequences are very different from each other, particularly in the region that binds to the receptor.

These viral envelope proteins are considered among the main targets to develop vaccines and antiviral therapies. In particular, for vaccine production, different strategies have been addressed. The proteins can be expressed on suitable vectors, like non-replicating adenovirus (Lasaro and Ertl, 2009). Another approach for vaccine production is based on the injection of the appropriate mRNA or DNA encoding the protein of interest into the host cells (Fynan et al., 1995). Production of the protein by the host machinery elicits the necessary immune response. However, strategies that involve production of Spike proteins, which can be considered the natural target of neutralizing antibodies, face several challenges.

In particular, it should be recalled that it may be very problematic to reconstruct proteins anchored to the membrane, since protein folding is a very complex process. Even small differences with the native environment can lead to conformational changes, which might elicit the production of antibodies that are not able to recognize the native structure of the target protein. Moreover, these proteins have a region exposed to the solvent that includes many domains, often interacting with each other, and this increases the complexity of the system.

In order to avoid the folding problem, an alternative approach could be to focus on short segments of the proteins. Short regions on the surface of the protein and exposed to the solvent can be recognized by the immune system.

## A Strategy Based on Loop Analysis

Rather than focusing on the entire protein, short regions may be considered potential candidates to elicit the immune response.

Loop regions, in particular, could be good candidates for such an approach, since they do not have a specific secondary structure (like helical or strand regions). Thus, we suggest focusing on protein fragments. In particular, we suggest an analysis of the amino acid sequences of loop regions, i.e., regions that do not possess a secondary structure, and which are located in solvent exposed areas that are recognizable by the immune system. Loop regions have the advantage that, lacking a defined secondary structure, they can be freely grafted since do not have specific conformational constraints. Loop regions can be easily identified from the 3D structure of the protein, or, if this is not available, they can be identified either by alignment with similar proteins of known structure (Edgar and Batzoglou, 2006) or by using prediction tools (Orry and Abagyan, 2012).

After identification of the loop regions, the next step is to recognize the ones exposed on the surface of the protein. Several tools are available to this purpose, either by looking at the 3D structure of the protein, when available, or by reconstructing the structure of the protein by homology modeling or by using prediction tools.

Finally, a search of the putative loops for similar sequences in the human genome allows select the best candidates to elicit an immune response. Several database search engines are available. This is an important step as well, in order to avoid using regions that would not be recognized as non-self by the immune system. The candidate sequences can be covalently linked to a selected support such as a polymer matrix to form a bioconjugate polymer. One of the best method for the development of this hybrid protein-polymer system is the use of crosslinker molecules (Ju et al., 2018; Murata et al., 2018) that allows to prepare site-specific polymer bioconjugates (Chen et al., 2020). Bio-conjugation that binds a single polymer chain to a synthon moiety distant from the active center was explored firstly for the pegylation route and developed in 2001 to other polymers (Veronese, 2001). The more suitable strategy to attach the selected target sequence to a polymer matrix is the “grafting to” method using a preformed polymer (Broyer et al., 2011). Polymers must joint different properties including biocompatibility, processability and stability. Biocompatible polyimides, polyamide, and polyurethanes meet many requirements together with their co-polymers such as polyamides and polyurethanes functionalized with phosphocoline (Teo et al., 2016; Alibeik and Sask, 2019). N-terminal and C-terminal groups of the matrixes make these compounds suitable to react with the selected target sequence forming amide bonds. To ensure that the target sequence preserves the same conformational variability as observed in the native protein, the distance between the N- and C-terminal extremes of the selected sequence in the bioconjugate polymer have to be identical to the distance observed in the native protein.

Such a strategy would avoid the folding problem of the protein.

In principle, such an approach does not require the 3D structure of the protein but only comparison of the amino acid sequence with similar sequences, the structure of which is already known, thus making the procedure very fast, an important feature in the case of a rapid diffusion of the infection.

Concerning SARS-CoV-2 virus, the 3D structure of the Spike protein in the prefusion state is available and we performed an analysis of its structure.

The secondary structure is reported in Figure 1A and shows the structured regions as well as the disordered ones.

Analysis of the loop regions was conducted by visual inspection of the 3D structure of Spike protein using Chimera (Pettersen et al., 2004), a molecular graphics software that indicates regions of secondary structure and allowed us to identify several regions exposed to the solvent. A subsequent Blast and PeptideSearch on the Uniprot database (The UniProt Consortium, 2019) allowed us to filter out regions present in human proteins thus leaving only regions that can be recognized as a foreign element by our immune system.

Regions 210–220, 455–472, 495–501, 527–537, 555–564, and 703–710 may represent good candidates for such a strategy. The first region belong to the N-terminal domain of the protein, the three following regions to the RBD domain, and the remaining two regions belong to the linker between the S1 and S2 domain. Their location onto the 3D structure is shown in Figure 1B. Figures 1C,D also report the corresponding regions of the MERS-CoV and SARS-CoV proteins, respectively. It must be noted that there is a better agreement with SARS-CoV than to MERS-CoV Spike protein regarding loops location. However, the same analysis could easily be applied *de novo* to MERS-CoV and SARS-CoV for a better definition of the loops, and a subsequent check of their presence in human proteins.

Here, the Blast and PeptideSearch tools indicated that the selected regions are only present in bacterial protein or in other bat coronavirus species and not in human proteins.

The aforementioned sequences were submitted to a further bioinformatics analysis to check their propensity to be unstructured. The PEP2D server (Singh et al., 2019) confirmed such hypothesis, suggesting that region 210–220 is the only one that has a slight propensity to assumes a helical fold, Table 1.

**TABLE 1:**
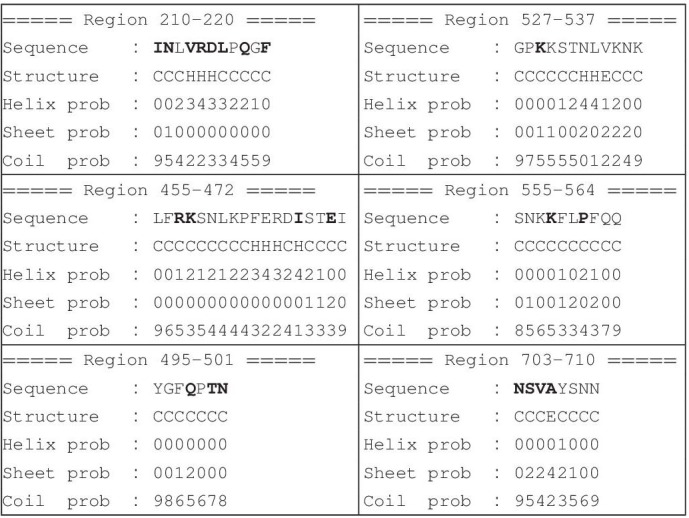
Secondary structure propensity of the reported regions evaluated by using the PEP2D server (Singh et al., 2019).

*The evaluated structure refer to C, Coil; H, Helix; E, Sheet. The residues that have been observed to mutate (Guruprasad, 2021) are in bold.*

Propensity of peptides to maintain an unordered structure can be conveniently checked by spectroscopic tools. We are actually working to a better definition of the boundaries of such regions and to check their propensity to maintain a disordered structure through circular dichroism or NMR spectroscopy.

It is important to note that the correct definition of the limits of the loop regions is critical for such an approach. In our case some quite long regions are not solved in the three-dimensional structure, or they represent loops with very short regions (2–3 amino acids) of secondary structure. In our analysis two structures of the Spike protein in the prefusion state were used (Melero et al., 2020; Wrapp et al., 2020; PDB codes 6zow and 6vsb, respectively), and the regions with ambiguities were discarded. Another approach would be to compare the amino acid sequence with the sequence of similar proteins having a solved high resolution 3D structure. Likewise, regions containing a cysteine have been discarded in our analysis, since this can forms a disulfide bridge that anchors the loop to the core of the protein thus giving a defined orientation. Finally, loop regions containing glycosylated amino acids could represent a problem as well.

## Discussion

A strategy based on grafting selected loop regions of antigenic proteins to suitable biopolymers has been described.

This strategy aims to avoid the production of antigenic proteins, which is inherently related to the correct folding of the proteins themselves. Conversely, short, unfolded regions are not subject to conformational constraints.

The concept of graft epitope peptides onto a polymeric matrix has been exploited linking peptides at one ending (Nuhn et al., 2013; Chandrudu et al., 2016; Skwarczynski and Toth, 2016). Also, epitope grafting onto selected proteins has been envisaged (Correia et al., 2011; Azoitei et al., 2012).

To the best of our knowledge, grafting loops representing potential epitopes trying to maintain their conformation by bridging their edge onto a polymeric matrix has not been considered yet.

These regions can be selected by inspecting the 3D structure of the protein or, in case of absence of the 3D structure, by comparing its amino acid sequence with the one of proteins whose structure is known.

Several bioinformatics tools can be used to predict potential epitopes and it has been pointed out that such tools can give different results (Lim et al., 2020). For such reason our analysis was performed by directly looking at regions lacking a secondary structure, not solvent exposed and not present in human proteins. Interestingly, some of the regions proposed with our analysis (i.e., regions 455–472, 495–501) are similar to the ones obtained by comparing the predicted results from different server (Lim et al., 2020). Another region, 703–710 is contiguous. It must be noted that in some cases the common regions obtained by comparing the results from different server contain secondary structure elements, and thus cannot overlap with our results.

It is important to study multiple target regions either to find the best region or to manage a combined use of multiple targets, each target in its own matrix. Being equipped with multiple targets is useful to face mutations of the protein.

Mutations in 400 different sites of the Spike protein have been reported (Guruprasad, 2021). The residues observed to mutate in the proposed regions are shown in bold in Table 1. Most of the residues of region 210–220, belonging to the N-terminal domain, have been reported to be mutated. The other regions may contain mutated residues as well, although to a lesser extent. In particular, mutations at residues 500 and 501 have been observed to be critical for the binding to ACE2 receptor. Thus, some of the reported mutations affect the proposed regions as well.

The presence of such mutations in these regions is not surprising since it is known that mutations mainly occur in the loop regions, given that they do not destroy the structural integrity of the protein. Therefore, in the case of a mutation in one of the loops employed, the antibodies generated in response to that specific target will not be effective. However, the simultaneous presence of antibodies generated in response to other targets would remove the negative effect of that mutation, allowing the immune system to recognize the mutated protein.

The immune-reactivity of the selected loops linked with the synthetic matrices and cocktails thereof should be investigated against the sera from COVID-19 patients taken at different times from the disease onset, from subjects that have been immunized with COVID-19 vaccines, and from controls taken in the pre-COVID-19 era, and results should be correlated with those obtained with commercial immunometric tests that use the Spike protein of SARS-CoV-2 as an antigen and with microneutralization tests. Furthermore, the use of these cocktails as immunogens in animals is envisaged, in order to verify the formation of neutralizing antibodies.

It is important to note that this approach provides that the matrix can accommodate different loops depending on the user choice thus making it a modular system that can be suitable for other pathogens as well. In particular, since the sequences of SARS-CoV and SARS-CoV-2 are very different from each other, but their three-dimensional structures and mechanism of action are very similar, it is possible to hypothesize to find analogs of the loop sequences and to be able to use such analogs in order to verify their reactivity toward known SARS antibodies or to possibly produce new ones. Furthermore, upon the appearance of a new unknown pathogen, a sequence analysis could lead to delineate potential loop regions of a membrane protein that can be quickly tested.

## Data Availability Statement

The original contributions presented in the study are included in the article/supplementary material, further inquiries can be directed to the corresponding author/s.

## Author Contributions

MD, GR, and MS conceptualized, drafted, and finalized the manuscript. All authors approved the submitted version.

## Conflict of Interest

The authors declare that the research was conducted in the absence of any commercial or financial relationships that could be construed as a potential conflict of interest.
